# Small signaling peptides define leaf longevity

**DOI:** 10.3389/fpls.2025.1616650

**Published:** 2025-06-20

**Authors:** Liping Qiu, Rong Lu, Ziling Zhang, Jiaxin Nie, Yue Wang, Jianping Liu, Huibin Han

**Affiliations:** ^1^ Jiangxi Engineering Laboratory for the Development and Utilization of Agricultural Microbial Resources, College of Bioscience and Bioengineering, Jiangxi Agricultural University, Nanchang, China; ^2^ Research Center of Plant Functional Genes and Tissue Culture Technology, College of Bioscience and Bioengineering, Jiangxi Agricultural University, Nanchang, China; ^3^ Jiangxi Province Key Laboratory of Vegetable Cultivation and Utilization, Jiangxi Agricultural University, Nanchang, China

**Keywords:** leaf senescence, CLE peptide, SCOOP peptide, MIK2, ROS

## 1 Introduction

Leaf senescence, the orchestrated degradation of cellular and tissue components that precipitates aging and eventual death, represents an adaptive mechanism allowing plants to efficiently reallocate resources and respond to fluctuating environmental conditions (Woo et al., 2019; Guo et al., 2021; Ahmad et al., 2024). The onset of senescence is marked by chlorophyll degradation, leading to leaf yellowing, a process driven by extensive metabolic reprogramming at various stages of senescence (Woo et al., 2019). Plants have developed intricate signaling networks to sense senescence-related cues, including abiotic and biotic stressors, age, and developmental signals. Consequently, an array of regulatory pathways, encompassing epigenetic modifications, (post) transcriptional, and (post) translational regulations, are activated (Woo et al., 2019; Guo et al., 2021; Zhang et al., 2021). Senescence-associated genes (SAGs) serve as pivotal key hubs in transmitting senescence signals, and their expression and function are regulated by multiple transcription factor (TF) families, such as WRKYs and NACs (Bengoa Luoni et al., 2019; Cao et al., 2023; Ahmad et al., 2024). However, the precise molecular mechanism underlying leaf senescence is still largely unexplored.

Phytohormones are pivotal in modulating leaf senescence and can be categorized into senescence promoters and retardants (Jibran et al., 2013; Guo et al., 2021; Asim et al., 2023). Besides these well-established roles of phytohormones, small signaling peptides have emerged as indispensable regulators in various aspects of plant developmental and adaptive processes (Xie et al., 2022; Ji et al., 2025; Xiao et al., 2025; Zhang et al., 2025). Typically composed of fewer than 100 amino acids, small signaling peptides are usually synthesized in the cytoplasm as prepropeptides, and they undergo processing or post-translational modifications in the endoplasmic reticulum (ER) and Golgi apparatus. Subsequently, they are transported to the apoplast, where they execute their physiological functions (Olsson et al., 2019). Then apoplast localized small signaling peptides are usually recognized by their specific membrane-bound receptors or co-receptors that usually belongs to the leucine-rich repeat receptor-like kinases (LRR-RLKs) family (Ji et al., 2025; Xiao et al., 2025; Zhang et al., 2025). The peptide-receptor module orchestrates either long-distance or local signaling cascades, thereby modulating developmental and adaptive responses through multiple regulatory mechanisms, including (post) transcriptional, (post) translational, and epigenetic modifications (Ji et al., 2025; Xiao et al., 2025; Zhang et al., 2025). Research has demonstrated that small signaling peptides from *Arabidopsis thaliana* such as CLAVATA3/EMBRYO-SURROUNDING REGION-RELATED (CLE) (Han et al., 2022; Zhang et al., 2022a, 2022b), SERINE-RICH ENDOGENOUS PEPTIDE (SCOOPs) (Zhang et al., 2024a), PHYTOSULFOKINE (PSK) (Yamakawa et al., 1999; Matsubayashi et al., 2006; Komori et al., 2009), and INFLORESCENCE DEFICIENT IN ABSCISSION-LIKE6 (IDL6) (Guo et al., 2022) are integral in managing leaf senescence by modulating distinct signaling pathways, thereby providing novel mechanistic insights into the regulation of leaf senescence.

## 2 CLE peptides delay leaf senescence via ethylene and ROS pathways

**Figure 1:**
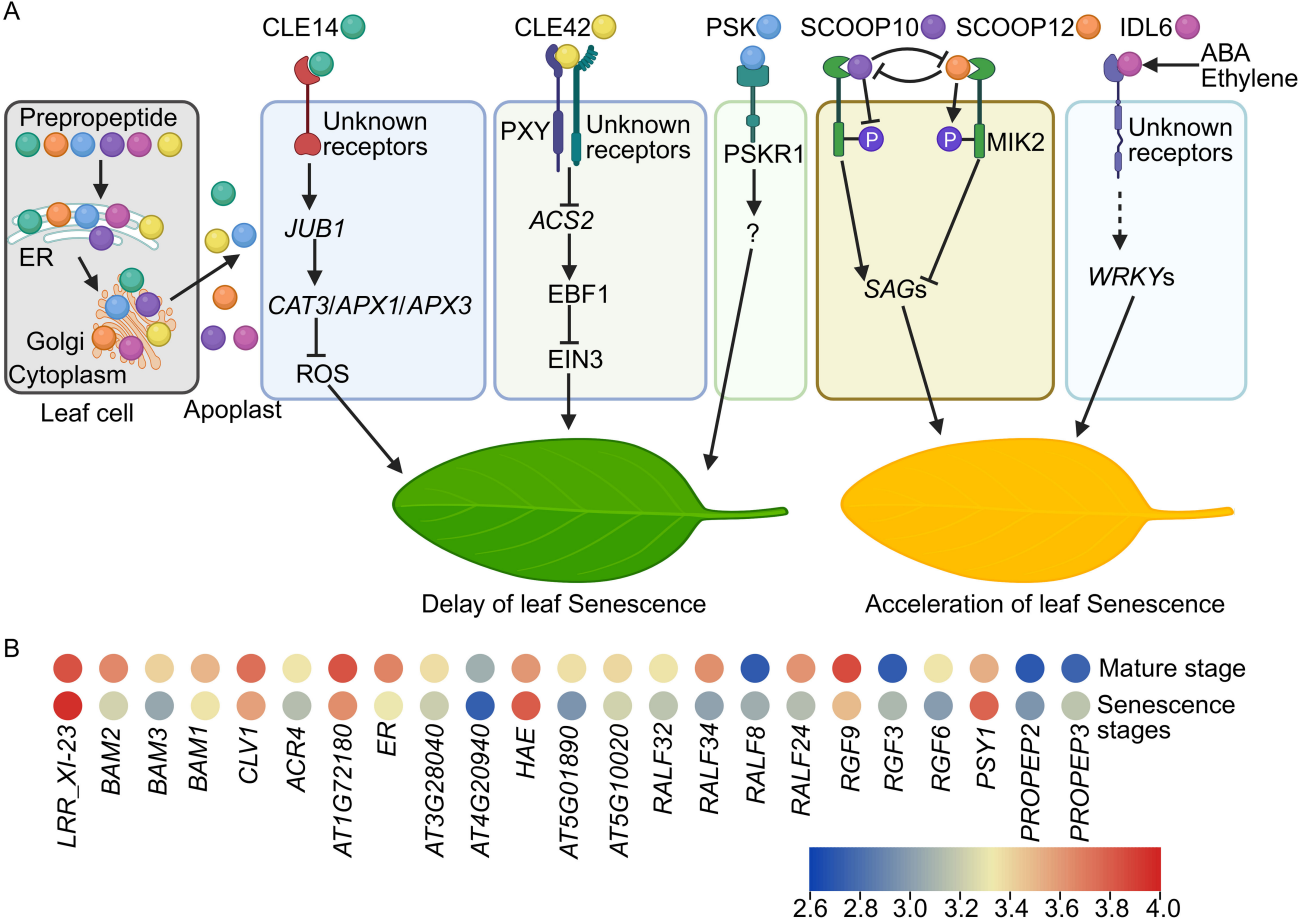
Small signaling peptides regulate leaf senescence. **(A)** In leaf cells, the leaf senescence associated small signaling peptides are synthesized in cytoplasm and undergo processing or post-translational modifications in the endoplasmic reticulum (ER) and Golgi apparatus. Subsequently, they are transported to the apoplast, where they execute their physiological functions. Unknown receptors detect the CLE14 signal, leading to the transcriptional activation of *JUB1* expression. *JUB1* subsequently enhances the transcription of ROS scavenging genes such as *CAT3*, *APX1*, and *APX3*, resulting in a reduction of ROS levels and a postponement of leaf senescence. CLE42 interacts with PXY and unidentified receptors to inhibit *ACS2* expression, thereby decreasing ethylene levels. The reduced ethylene content induces the accumulation of EBF1 proteins, which disrupt the function of EIN3 and ethylene responses, ultimately delaying leaf senescence. PSKR1 recognizes the PSK peptide signal to delay leaf senescence via undefined mechanisms. SCOOP10 and SCOOP12 peptides antagonistically regulate leaf senescence in a MIK2-phosphorylation dependent manner. During the early stage of leaf senescence, the SCOOP10 peptide inhibits the biosynthesis of the SCOOP12 peptide. Subsequently, SCOOP10 directly binds to the receptor MIK2, inhibiting its phosphorylation and induces the *SAG*s expression, thereby promoting the senescence process. At the later stages, *PROSCOOP12* is translated and processed into the SCOOP12 peptide. The SCOOP12 peptide then outcompetes the binding of SCOOP10 with MIK2, facilitating MIK2 phosphorylation and suppresses the *SAG*s expression, consequently delaying leaf senescence. The IDL6 peptide modulates leaf senescence via transcriptional regulation of WRKY TFs through unidentified receptors. Abscisic acid (ABA) and ethylene also activate IDL6 signaling to influence leaf senescence. **(B)** Expression profiles of genes encoding *LRR*-*RLK*s and *RAPID ALKALINIZATION FACTOR*s (*RALF*s), *PLANT PEPTIDE CONTAINING SULFATED TYROSINE1* (*PSY*1), *ROOT MERISTEM GROWTH FACTOR*s (*RGF*s), and *ELICITOR PEPTIDE PRECURSOR*s (*PROPEP*s). Data is sourced from Lyu et al., 2019, and the heatmap is generated using TBtools (Chen et al., 2023b) with the average log FPKM values. P: phosphorylation. Dashed line means indirect regulations.

CLE proteins generally possess an N-terminal signal sequence that guides them into the secretory pathway, a central variable domain, and one or multiple conserved CLE motifs at the C-terminus, which are typically post-translationally modified to produce functional polypeptides (Fletcher, 2020; Xie et al., 2022). Transcriptomic analyses indicate differential expression of *CLE* genes in mature and senescent leaves, implying their involvement in leaf senescence (Lyu et al., 2019; Han et al., 2022). Specifically, CLE14 and CLE42 peptides are crucial in delaying leaf senescence (Zhang et al., 2022a, 2022b). The expression level of *CLE14* and *CLE42* is induced by multiple senescence clues, such as salinity, drought, and darkness (Zhang et al., 2022a, 2022b). Mutants deficient in *CLE14* or *CLE42* gene function exhibit early leaf senescence, whereas transgenic plants overexpressing *CLE14* or *CLE42* genes show delayed senescence (Zhang et al., 2022a, 2022b). Exogenous application of synthetic 12-amino-acid CLE motifs can mimic the endogenous functions of CLE peptides (Zhang et al., 2019; Kang et al., 2022). Similarly, leaves treated with synthetic CLE14 or CLE42 peptides also display a delayed senescence phenotype (Zhang et al., 2022a, 2022b). Notably, CLE14 and CLE42 peptides activate distinct signaling pathways to modulate leaf senescence (**Figure 1A**) (Zhang et al., 2022a, 2022b). CLE14 peptide upregulates the expression of *JUNGBRUNNEN1* (*JUB1*), a NAC family transcription factor, which in turn enhances the expression of reactive oxygen species (ROS) scavenging genes, thereby reducing ROS levels and delaying senescence (Zhang et al., 2022a). Conversely, CLE42 peptide downregulates the expression of ACC synthases (ACSs), key enzymes in ethylene biosynthesis, resulting in lower ethylene levels (Zhang et al., 2022b). The decreased ethylene level in leaves leads to the accumulation of EIN3-BINDING F-BOX (EBF) proteins, which mediate the degradation of ETHYLENE-INSENSITIVE3 (EIN3) protein via the proteasome pathway (Guo and Ecker, 2003), thereby impairing EIN3 function and ethylene responses, ultimately delaying leaf senescence (**Figure 1A**). The LRR-RLK PHLOEM INTERCALATED WITH XYLEM (PXY) partially transmits CLE42 signal to regulate leaf senescence. Overall, CLE peptides modulate leaf senescence through distinct signaling mechanisms (**Figure 1A**) (Han et al., 2022; Zhang et al., 2022a, 2022b).

## 3 SCOOP peptides antagonistically regulate leaf senescence

SCOOPs are classified into the phytocytokine peptide family. The precursors of SCOOPs, known as PROSCOOPs, undergo proteolytic processing at the N-terminus to yield the bioactive C-terminal SCOOP peptides (Gully et al., 2019). In *Arabidopsis thaliana* genome, over 50 SCOOP peptide members have been identified (Yang et al., 2023), and they play pivotal roles in plant immune responses (Gully et al., 2019; Hou et al., 2021; Rhodes et al., 2021; Stahl et al., 2022; Jia et al., 2024; Wu et al., 2024), root development (Guillou et al., 2022a; Wang et al., 2024), flowering timing (Guillou et al., 2022b), and leaf senescence (Zhang et al., 2024a; Brusslan, 2025). *PROSCOOP* expression varies at different stages of leaf development, with *PROSCOOP10* showing upregulated expression at early senescence stage, while *PROSCOOP12* being markedly upregulated in later senescence stages, indicating their roles in leaf senescence process (Zhang et al., 2024a). Mutations in *PROSCOOP10* results in delayed leaf senescence, while exogenous application of synthetic SCOOP10 peptide induces premature senescence (Zhang et al., 2024a). Furthermore, overexpression of *PROSCOOP10* similarly promotes premature senescence. Conversely, application of synthetic SCOOP12 peptide or overexpression of *PROSCOOP12* delays senescence, suggesting antagonistic functions of SCOOP10 and SCOOP12 peptides in leaf senescence regulation (Zhang et al., 2024a).

The LRR-RLK receptor, MALE DISCOVERER 1-INTERACTING RECEPTOR-LIKE KINASE 2 (MIK2), has been identified as a receptor for SCOOP10 and SCOOP12 peptides (Hou et al., 2021; Rhodes et al., 2021). *MIK2* is predominantly expressed in senescing leaves. The *mik2* mutant exhibits accelerated senescence, while *MIK2* overexpression transgenic lines show delayed senescence, indicating that MIK2 is crucial for leaf senescence (Zhang et al., 2024a). Microscale thermophoresis (MST) assays corroborate the competitive binding of SCOOP10 and SCOOP12 peptides to MIK2 receptor. Further investigations reveal that SCOOP10 peptide inhibits MIK2 phosphorylation, whereas SCOOP12 peptide enhances MIK2 phosphorylation. Additionally, SCOOP12 peptide suppresses the expression of *SAG*s-induced and MIK2 phosphorylation by SCOOP10 peptide. Collectively, SCOOP12 peptide antagonizes SCOOP10 peptide by modulating MIK2 phosphorylation and senescence signaling pathways during late senescence stages, thereby finely regulating the leaf senescence process (**Figure 1A**) (Zhang et al., 2024a).

## 4 PSK and IDA peptides participate in leaf senescence regulation

PSKs constitute a group of disulfated pentapeptides, encompassing four bioactive variants: PSK-α, -γ, -δ, and -ϵ. These peptides are perceived by plasma membrane-localized receptors, known as PSK RECEPTORs (PSKRs), to modulate various physiological processes including cellular proliferation and expansion, plant reproduction, somatic embryogenesis, regeneration, legume nodulation, leaf senescence, and stress resilience against biotic and abiotic clues (Yamakawa et al., 1999; Matsubayashi et al., 2006; Li et al., 2024). Exogenous application of the PSK-α peptide has been observed to delay leaf senescence, potentially by regulating chlorophyll integrity (**Figure 1A**) (Yamakawa et al., 1999). Mutation of PSKR receptor accelerates the senescence process (Matsubayashi et al., 2006). Nonetheless, conflicting evidence exists concerning the involvement of PSKR1 receptors in leaf senescence (Matsubayashi et al., 2006; Yadav et al., 2024). Crucially, the bioactivation of PSK peptides necessitates tyrosine sulfation, catalyzed by the transmembrane enzyme tyrosylprotein sulfotransferase (TPST). Consequently, a loss-of-function mutation in TPST precipitates premature leaf senescence, mirroring the effects observed with PSK peptide application (Yamakawa et al., 1999; Matsubayashi et al., 2006; Komori et al., 2009).

The IDA/IDL peptides, initially identified for their critical role in organ abscission, are also implicated in various biological processes, including responses to biotic and abiotic stress (Wang et al., 2023). *IDL6* transcription is markedly upregulated in leaves during both early and late senescence stages, indicating its involvement in leaf senescence (Guo et al., 2022). The *idl6* loss-of-function mutant exhibits a pronounced delay in leaf senescence, and this delayed senescence phenotype can be reversed by reintroducing the *IDL6* gene into *idl6* mutant plants. In contrast, leaves overexpressing *IDL6* or treated with exogenous synthetic IDL6 peptide display an early senescence phenotype. Transcriptomic analysis reveals that *WRKY53*, *WRKY38*, and *WRKY62* TFs may act downstream of IDL6 in promoting leaf senescence. Additionally, IDL6 may also play a role in abscisic acid (ABA) and ethylene-mediated acceleration of leaf senescence (**Figure 1A**) (Guo et al., 2022).

## 5 Future perspectives

Leaf senescence represents an essential evolutionary strategy that enhances plant fitness and survival by facilitating nutrient remobilization to support the growth of sink organs, such as roots, stems, and flowers (Woo et al., 2019; Guo et al., 2021; Ahmad et al., 2024). While these studies have elucidated the intricate roles of small signaling peptides in leaf senescence (**Figure 1A**), several unresolved questions remain to be explored in future researches. The answers to these questions will accelerate the application of small signaling peptides in agriculture to recycle of the nutrients.

1. Characterization of novel small signaling peptides in leaf senescence. The expression level of several small signaling peptide genes, such as *RAPID ALKALINIZATION FACTOR*s (*RALF*s), *PLANT PEPTIDE CONTAINING SULFATED TYROSINE1* (*PSY1*), *ROOT MERISTEM GROWTH FACTOR*s (*RGF*s), and *ELICITOR PEPTIDE PRECURSOR*s (*PROPEP*s) are also regulated during senescence (**Figure 1B**) (Lyu et al., 2019), indicating the presence of unidentified small signaling peptides involved in the regulation of leaf senescence. Mass spectrometry (MS) is a reliable method to identify and verify most peptide members in plants. However, MS has limitations in detecting low-abundance peptides in plants. Mass spectrometry imaging (MSI) techniques offer advanced capabilities with superior sensitivity and high spatial resolution, enabling the visualization of the spatial distribution of small peptides at various stages of leaf senescence, even at single-cell resolution (García-Rojas et al., 2024; Petřík et al., 2024; Zhang et al., 2024b). Integrating MSI with MS techniques will facilitate the identification of previously uncharacterized small signaling peptides involved in leaf senescence.
2. How to maintain the homeostasis of small signaling peptides during leaf senescence? Plants synthesize a multitude of small signaling peptides (Xie et al., 2022; Ji et al., 2025; Xiao et al., 2025; Zhang et al., 2025) as well as noncanonical peptides (NCPs) (Wang et al., 2020; Pei et al., 2022; Sami et al., 2024). These peptides appear to play synergistic or antagonistic roles in leaf senescence (**Figure 1A**), although their interactions in leaf senescence are not clear. Therefore, it is crucial to understand how plants precisely regulate the levels of these small signaling peptides to achieve optimal cellular responses to senescence cues. Notably, the specific function of NCPs in the process of leaf senescence necessitates additional in-depth investigation in future. In addition, the application of PSK peptide has been shown to delay the senescence of fruits (Aghdam et al., 2021a) and cut flowers (Aghdam et al., 2021b), indicating a conserved regulatory function of PSK peptide in senescence mechanisms. Remarkably, numerous homologs of these senescence-associated small signaling peptides have been identified across various plant species (Ji et al., 2025; Zhang et al., 2025). Nevertheless, their biological roles in the modulation of senescence processes in other plant species remain to be elucidated.
3. Identification of novel receptors. Typically, plasma membrane localized LRR-RLK receptors are capable of perceiving small signaling peptides, thereby modulating an array of signaling pathways (Furumizu and Aalen, 2023; Ji et al., 2025; Xiao et al., 2025; Zhang et al., 2025). A couple of LRR-RLKs encoding genes, such as *BARELY ANY MERISTEM*s (*BAM*s) and *CLAVATA1* (*CLV1*) are (de)activated in senescent leaves (**Figure 1B**), implying that these receptors might convey CLE, SCOOP, IDL6, or PSK signals to regulate leaf senescence. But their roles in leaf senescence requires further investigations. Moreover, 4-azidosalicylic acid-labeled peptides and CRISPR-based genetic screening systems present opportunities for the identification of novel receptors specific to leaf senescence-related small signaling peptides, with high specificity and throughput (Shinohara and Matsubayashi, 2017; Gaillochet et al., 2021). Additionally, various *in vitro* analytical techniques, employing either labeled or label-free ligands, can be utilized to validate interactions between small signaling peptides and their corresponding receptors (Sandoval and Santiago, 2020).
4. Construction of regulatory networks at the (post)transcriptional and (post)translational levels. As mentioned, the intricate signaling pathways involved in small signaling peptides-mediated leaf senescence regulation remain largely elusive (**Figure 1A**). Recently, a comprehensive single-cell RNA sequencing (scRNA-seq) transcriptomic analysis has facilitated the identification of pivotal hub genes that governs leaf senescence (Guo et al., 2025). Spatial transcriptomic technologies enable the precise localization and quantification of spatial gene expression across various plant tissues and developmental stages (Yin et al., 2023; Sang and Kong, 2024). These advanced RNA-seq methodologies will uncover differentially expressed gene clusters that specifically respond to leaf senescence-related small signaling peptides. Post-translational modifications (PTMs) of proteins, including acetylation, crotonylation, glycosylation, lysine lactylation, methylation, phosphorylation, SUMOylation, and ubiquitylation, are ubiquitous in diverse biological processes, ensuring rapid and tight regulation of signal transduction and cellular responses during leaf senescence (Woo et al., 2019; Zhang et al., 2021; Guo et al., 2021). The advent of 4D proteomics (Chen et al., 2023a; Hao et al., 2023) allows for in-depth proteomic exploration with high speed, robustness, sensitivity, and selectivity. This technique will offer crucial insights into protein abundance, stability, and post-translational modifications in leaf senescence (Han et al., 2022). Furthermore, epigenetic regulation plays a vital role in leaf senescence (Ostrowska-Mazurek et al., 2020; Zhang et al., 2021; Miryeganeh, 2022; Jeong et al., 2025). CRISPR-based epigenetic tools, such as CRISPR interference (CRISPRi), CRISPR/dCas9 activation (CRISPRa), and CRISPR-dCas9-DNMT3A (Jogam et al., 2022; Liu et al., 2022; Qi et al., 2023), can be employed to investigate the effects of small peptides on senescence-related gene expression and epigenetic regulations. In summary, leveraging advanced RNA-seq and proteomic technologies will facilitate the construction of unprecedented transcriptional and protein networks mediated by small signaling peptides that control leaf senescence.
5. How small signaling peptides integrate phytohormones and environmental cues. Leaf senescence can be triggered by various abiotic factors such as light, circadian rhythms, drought, salinity, nitrogen deprivation, and high temperatures (Woo et al., 2019; Tan et al., 2023; Wang et al., 2025; Vander Mijnsbrugge et al., 2025). The reported data primarily elucidated the biological functions of these small signaling peptides in the regulation of age-dependent leaf senescence. Importantly, the transcriptional levels of *CLE14/CLE42, IDL6, PROSCOOP10/12* were induced in response to environmental stressors associated with senescence, such as drought, salinity, and darkness (Guo et al., 2022; Zhang et al., 2022a, 2022b; Zhang et al., 2024a). This suggests that senescence associated small signaling peptides may be involved in stress-induced leaf senescence, although further research is warranted to confirm their interactions. Moreover, phytohormones are pivotal in modulating leaf senescence via intricate interactions (Guo et al., 2021; Zhang et al., 2021; Huang et al., 2022). Notably, small signaling peptides are implicated in the response to phytohormones (Morcillo et al., 2024; Wang et al., 2024; Chen et al., 2025; Ji et al., 2025; Mou et al., 2025). This may indicate that senescence associated small signaling peptides may also serve as crucial integrators to link with hormonal pathways to regulate leaf senescence. Nonetheless, the precise mechanisms remain to be elucidated.
